# Experimental and Numerical Investigation of Acoustic Emission Source Localization Using an Enhanced Guided Wave Phased Array Method

**DOI:** 10.3390/s24175806

**Published:** 2024-09-06

**Authors:** Jiaying Sun, Zexing Yu, Chao Xu, Fei Du

**Affiliations:** 1 School of Astronautics, Northwestern Polytechnical University, Xi’an 710072, China; sun_jiaying@mail.nwpu.edu.cn (J.S.); yuzexing@mail.nwpu.edu.cn (Z.Y.); dufei@nwpu.edu.cn (F.D.); 2 Yangtze River Delta R&D Institute, Northwestern Polytechnical University, Suzhou 215400, China

**Keywords:** guided wave phased array, acoustic emission, source localization, automatic wave velocity calculation, structural health monitoring

## Abstract

To detect damage in mechanical structures, acoustic emission (AE) inspection is considered as a powerful tool. Generally, the classical acoustic emission detection method uses a sparse sensor array to identify damage and its location. It often depends on a pre-defined wave velocity and it is difficult to yield a high localization accuracy for complicated structures using this method. In this paper, the passive guided wave phased array method, a dense sensor array method, is studied, aiming to obtain better AE localization accuracy in aluminum thin plates. Specifically, the proposed method uses a cross-shaped phased array enhanced with four additional far-end sensors for AE source localization. The proposed two-step method first calculates the real-time velocity and the polar angle of the AE source using the phased array algorithm, and then solves the location of the AE source with the additional far-end sensor. Both numerical and physical experiments on an aluminum flat panel are carried out to validate the proposed method. It is found that using the cross-shaped guided wave phased array method with enhanced far-end sensors can localize the coordinates of the AE source accurately without knowing the wave velocity in advance. The proposed method is also extended to a stiffened thin-walled structure with high localization accuracy, which validates its AE source localization ability for complicated structures. Finally, the influences of cross-shaped phased array element number and the time window length on the proposed method are discussed in detail.

## 1. Introduction

Acoustic emission is a phenomenon of radiation of acoustic (elastic) waves in solids that occurs when a material undergoes irreversible changes in its internal structure [1,2]. In particular, AE occurs during the processes of mechanical loading of materials and structures accompanied by structural changes that generate local sources of elastic waves [3,4]. This results in small surface displacements of a material produced by elastic or stress waves generated when the accumulated elastic energy in a material or on its surface is released rapidly [5,6]. Therefore, every occurrence of an AE event represents potential structural damage and localizing the AE source is of great importance to maintain the safety and reliability of the structure. For thin-walled structures, guided waves caused by AE can be captured by in situ bonded or embedded smart sensors, such as piezoelectric sensors [7,8] and fiber optic sensors [9,10]. By properly arranging transducers and combining them with appropriate algorithms, the localization of the AE source can be accurately determined. Therefore, exploiting an AE-generated guided wave and using it for damage detection is a potential direction for structural health monitoring (SHM) [11,12,13].

Generally, the acoustic emission detection method uses a sparse sensor array to identify the damage and its location [14]. The most popular method of AE source localization is the triangulation technique [15], which has been commercialized nowadays. The method uses at least three sensors to solve the location of the AE source. Basically, the principle is to use the arrival time together with the wave velocity to determine the sensor-source distance, combining several pieces of distance information to obtain the location of the AE source. However, it is noted that for a commercial AE detection system, special heavy AE sensors and a pre-defined wave velocity are often necessary, and the localization accuracy is largely affected by the prior knowledge of wave velocity and signal quality recorded by the sparse sensor array. The strategy based on time-reversal (T-R) processing is another efficient way to detect the AE source in thin-walled structures [16,17]. For T-R-based AE localization [18], the transducers record the signals when an AE event occurs. The responses of all channels are reversed in the time domain and reapplied as the input signals to the respective transducers. According to the spatial focus property of T-R processing, the re-emitted wavefield generated by the transducers will focus on the AE source. This strategy does not require prior knowledge of the excitation profile and can avoid the adverse effects induced by the dispersion and reflection of waves [19]. However, in practice, the application of the T-R processing to locate the AE source involves two critical techniques: one is the full-field measurement of the wavefield in the monitoring area, and the other is the identification of the focus of the wavefield caused by the re-emitted T-R signals. The above procedures limit the wide use of this method in actual engineering. To tackle these problems, Yu et al. [18,20] proposed the virtual T-R method and applied it to stiffened plate and composite plate, respectively; the AE source localization and force reconstruction are all realized. However, this method requires the establishment of a high-fidelity numerical model of the application object in advance, which is relatively time consuming.

The guided wave phased array (GWPA) method, which is a dense sensor array method with a high signal-to-noise ratio, is considered a promising technique for detecting damage [21]. The biggest advantage of GWPA is that it can maintain better localization accuracy in a high-noise environment, which is vital for practical service conditions. Specifically, the active GWPA method [22,23] can scan and focus the wavefront at a user-defined direction in the structure, by manipulating the time delay between excitation signals emitted by each piezoelectric element. Then, GWPA elements receive the scattered wave reflected from damage and the response signals are synchronized by applying a certain time delay. As a result, the superposition signal will be maximum in the damage direction and the location will be easily solved by Time of Flight (TOF) and wave velocity.

For the GWPA method, transducer configuration has an important influence on the localization results. Linear array GWPA has been widely explored and investigated due to its simple configuration. Yu and Giurgiutiu [24] first applied phased array technology to a linear array of piezoelectric elements and successfully detected the crack damage in an aluminum plate. Yan and Rose [25] introduced a linear phased array in composite plates and demonstrated that GWPA can be implemented into anisotropic plates after careful selection of the guided wave mode and frequency range. However, the linear GWPA method was found to perform poorly at the angles close to the axes of the linear array and generated ambiguity (mirror effect). Then, some researchers proposed to adopt a 2-D array configuration to overcome the above-described issues. Giurgiutiu and Bao [26] proposed several improved array designs and used a rectangular array to illustrate the calculation procedure of a 2-D array configuration. Malinowski et al. [27] proposed a star-shaped array and verified its damage detection efficiency, but its configuration and post-processing algorithm are relatively complicated. Wang et al. [28] combined the cross-shaped GWPA with image enhancement to locate damage accurately, which achieves high efficiency with fewer elements and less complexity. Hence, it has been proven that the cross-shaped phased array can better balance the complexity of the algorithm and the accuracy of the result among other configurations.

Compared to applying GWPA method in active SHM, using GWPA as a passive method for AE source localization is distinctive. For such applications, the acoustic source is considered as the wave source. Then, phased array elements will receive the signals generated from the AE source. The response signals will be synchronized by applying a certain time delay. As a result, the superposition signal will be at its maximum in the AE source direction. The principle of using the phased array algorithm to locate acoustic emission sources is the same as the beamforming (delay-and-sum) algorithm. The main difference is that the phased array method is a dense sensor array method, and the distance between adjacent sensors is generally small. Beamforming does not clearly stipulate the distance between adjacent sensors. Beamforming is a signal processing technique used in sensor arrays for directional signal transmission or reception [29]. Beamforming is used in acoustic source localization, for which several localization algorithms have been developed. For AE source localization, a delay-and-sum algorithm is usually used. McLaskey et al. [30] first applied this method to AE source localization and only obtained the polar angle of AE source. He et al. [31] investigated the dispersion behavior of AE waves and its impact on the accuracy of the beamforming approach. In addition, they also investigated an AE source localization method based on near-field assumption using linear array GWPA [32], which performs well in identifying the AE source in the near-field of the array. However, since the response signals will only be recorded when the reference channel exceeds the trigger threshold, the real value of TOF (the time taken to travel from the AE source to the array) is hard to know. Therefore, it is hard to solve the location of AE source, and this makes the passive damage localization challenging. To tackle this challenge, Xiao et al. [33] investigated two linear arrays distributed in the x and y directions, respectively, and used arrays in different orientations to determine the corresponding coordinates, respectively. While this method is still a linear array method, for actually using one array to determine only one coordinate at a time, the localization accuracy is not very good. In addition, Nakatani et al. [34] found that the sensors are suggested to be placed as close as possible to improve the localization accuracy. This improvement in sensor placement is consistent with the sensor layout characteristics of the phased array method, which provides a theoretical basis for applying the phased array method to acoustic emission source localization.

At present, the acoustic emission source localization methods with an unknown wave velocity can be roughly divided into three categories. One is based on the Time of Arrival (TOA) or ΔTOA between sensors and special geometric configurations [35,36], or based on the normal configuration to perform an optimization algorithm [37,38]. The other is the time-reversal method [16,17]. Whether for the traditional or virtual T-R method, wave velocity is not required as a priori information. The third category is based on data-driven and machine learning that has gradually emerged in recent years [39,40]. However, among the above methods, the first method combines geometric information with optimization algorithms to accurately solve the AE sources and wave velocity, the optimization algorithm is a little computationally intensive. The machine learning-based and virtual time-reversal method both require the establishment of a complete database in advance, which is labor-intensive and time-consuming, while the traditional time reversal requires the monitoring of the entire wave field, which is difficult to apply in practice.

In summary, there still exist some challenges to applying GWPA in passive AE source localization. Firstly, the passive GWPA must record response signals when the reference channel exceeds the trigger threshold. Therefore, it is difficult to obtain TOF directly. Furthermore, it is hard to solve the location of the AE source through TOF and wave velocity. Secondly, the structures will be affected by various environmental and thermal loads in-service, resulting in the variation of guided wave velocity. However, past GWPA methods adopt a pre-defined velocity for beamforming, thus introducing errors in the polar angle-calculating step. Thirdly, the GWPA configurations for AE source localization are mostly linear arrays. Linear configuration may result in ambiguity symmetric to the array, which is an inherent geometrical limitation and is difficult to overcome through array optimization. To tackle the above problems, an enhanced guided wave phased array technique is developed in this paper. The proposed method is composed of a cross-shaped phased array and four additional far-end sensors to determine the exact location of AE sources. An automatic wave velocity determination method is proposed to solve the real-time wave velocity, which eliminates the need for pre-defined wave velocity. The radial distance along the decided polar angle is accurately solved with the automatically calculated wave velocity and enhanced information from far-end sensors. This paper is organized as follows. In Section 2, the theoretical background and algorithm procedure of the enhanced phased array method are introduced. In Section 3, the numerical model is established, then result validation is proposed. In Section 4, the experimental setup is illustrated. The proposed method is validated by comparing the localization accuracy with the classical triangulation technique. The proposed method is also extended to a stiffened thin-walled structure to validate its AE source localization ability for complicated structures. In Section 5, the influences of the array element number and time window length are discussed. In the last section, some conclusions are drawn.

## 2. The Proposed AE Source Localization Method

### 2.1. Enhanced Sensor Array Layout

**Figure 1:**
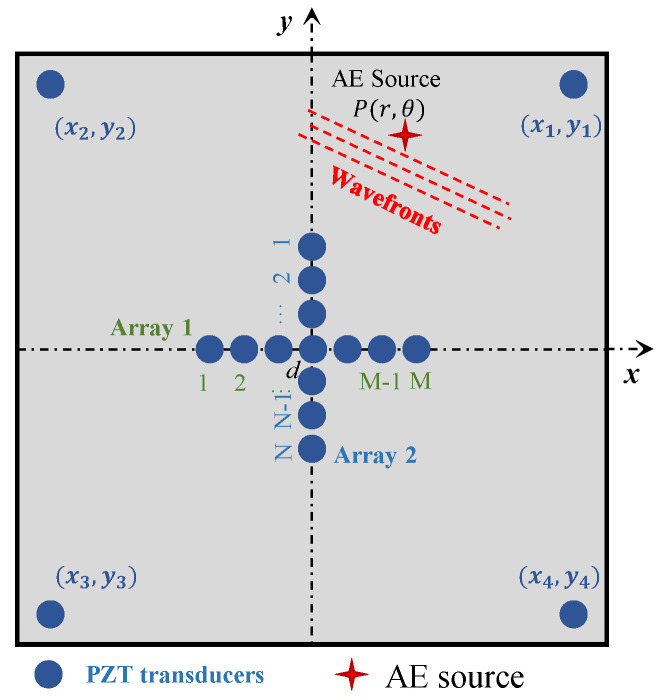
Layout of the enhanced phased array.

The array layout of the proposed localization method is shown in Figure 1. A cross-shaped phased array is arranged in the center of the coordinate system. The cross-shaped phased array is composed of two perpendicular linear arrays. The arrays are placed along the *x* and *y* axes, with the same element amount *M* and adjacent element spacing d, and the mid-point of both arrays is the axes’ origin. Since it is difficult to obtain TOF directly for passive GWPA, the radial distance of the AE source cannot be solved directly using TOF and wave velocity. Therefore, four far-end transducers are placed to provide enhanced signals and the conventional triangulation technique is modified to better localize the AE source. Far-end transducers are placed far away from cross-shaped phased array and the coordinates are $S_{1}\left(x_{1},y_{1}\right)$, $S_{2}\left(x_{2},y_{2}\right)$, $S_{3}\left(x_{3},y_{3}\right)$, $S_{4}\left(x_{4},y_{4}\right)$, respectively.

In practice, the acoustic emission point P(r,θ) can be considered as a wave source; thus, the AE signals will be received by the array elements, respectively. In the proposed method, acoustic emission localization is divided into two steps. The first step is to use an iterative method to solve the wave velocity and polar angle of the AE sources simultaneously. The second step is to determine the distance between the acoustic emission location and the coordinate’s origin.

### 2.2. Polar Angle Determination with Automatic Wave Velocity Calculation

In plate-like structures, AE sources excite guided ultrasonic waves (Lamb waves in particular) and the propagation velocities of these waves are a function of frequency. Hence, assuming that the wave velocities are constant in all directions and using the pre-defined velocity to localize the AE source is not adequate. To obtain the wave velocity corresponding to each AE source, automatically calculating wave velocity is necessary. However, the processes of calculating wave velocity and polar angle are coupled; the specific reasons are explained below. The wave velocity cannot be calculated without knowing the polar angle, and vice versa. Therefore, an iterative method is proposed to calculate the velocity and polar angle of the AE source simultaneously in step 1.

Using the concepts of antenna theory [41], the far-field can be defined as shown in Equation (1). When the AE source is located in the far-field, the wavefronts can be considered as straight lines. Similar to most AE source localization methods, the proposed AE source localization method is based on the far-field assumption. Hence, the propagation directions of the wave rays emitted from the AE source become approximately parallel. In this paper, due to the complexity of the AE signals (wideband, multi-mode), the Continuous Wavelet Transform (CWT) is performed on a raw AE signal using the AGU-Vallen wavelet program [42]. The mother wavelet used in the software (R2019.0926.2) is a Gabor wavelet based on the Gaussian function, as shown in Equation (2). In this way, the CWT coefficient of AE signals is used as the input in the following methods:

(1) $$R_{far}>2D^{2}/\lambda$$

where *D* is the total length of the array and λ is the wavelength.

(2) $$\psi \left(t\right)=\pi^{-1/4}{\left(\frac{\omega_{p}}{\gamma }\right)}^{1/2}exp\left[-\frac{t^{2}}{2}{\left(\frac{\omega_{p}}{\gamma }\right)}^{2}+i\omega_{p}t\right]$$

where $\omega_{p}$ is the center frequency and γ is a constant taken as $\gamma =\pi {\left(2/ln2\right)}^{1/2}=5.336$.

Using the phased array beamforming algorithm, the polar angle of acoustic emission source can be decided using cross-shaped phased array transducers, as shown in Figure 1. In the algorithm, signals are shifted in a certain time delay, related to the spacing from each transducer to the origin transducer and the beamforming angle ϑ. It is assumed that the AE source is located at the angle ϑ (0–360°); then, the corresponding time delay $\Delta t_{m}$ or $\Delta t_{n}$ for each sensor can be calculated, as shown in Equations (3) and (4). All the shifted AE signals will be summed up, as shown in Equation (5). If the angle ϑ is not the polar angle of the actual AE source, the amplitude of the superimposed signal will be small, while if the angle ϑ is the polar angle of the actual AE source, the amplitude of the superimposed signal will be the largest. As a result, the amplitude of the superimposed wave will be at its maximum in the angular coordinate of the AE source, as shown in Equation (6).

(3) $$\Delta t_{m}=d\left(m-\frac{\left(M+1\right)}{2}\right)\frac{cos\vartheta }{c}\;\;\;(m=1,\;2,\ldots ,M)$$

(4) $$\Delta t_{n}=d\left(n-\frac{\left(N+1\right)}{2}\right)\frac{sin\vartheta }{c}\;\;\;(n=1,\;2,\ldots ,N)$$

where *d* is the distance between two adjacent array elements, *M* is the elements amount of Array 1, *N* is the elements amount of Array 2, c is the wave group velocity. The superimposed wave amplitudes $S_{P}$ at the certain polar angle ϑ are shown in Equation (5), and the localized angular coordinate of the AE source is shown in Equation (6):

(5) $$S_{P}\left(t\right)=\sum_{m=1}^{M}\frac{A_{m}}{\sqrt{r_{m}}}S_{0}\left(t-\Delta t_{m}\right)+\sum_{n=1}^{N}\frac{A_{n}}{\sqrt{r_{n}}}S_{0}\left(t-\Delta t_{n}\right)\;\;=\sum_{m=1}^{M}S_{m}\left(t-\Delta t_{m}\right)+\sum_{n=1}^{N}S_{n}\left(t-\Delta t_{n}\right)$$

(6) $$\theta ={\left.\vartheta \right|}_{S_{p}=max\left(S_{\vartheta }\right)}$$

where $S_{0}$ is the original waveform, $A_{m}$ is the amplitude of mth element, $1/\sqrt{r_{m}}$ represents the attenuation of waveform amplitude, $S_{m}$ is the signal received from *m*th element, $A_{n}$ is the amplitude of nth element, $1/\sqrt{r_{n}}$ represents the attenuation of waveform amplitude, $S_{n}$ is the signal received from nth element, and θ is the localized angular coordinate of the AE source.

**Figure 2:**
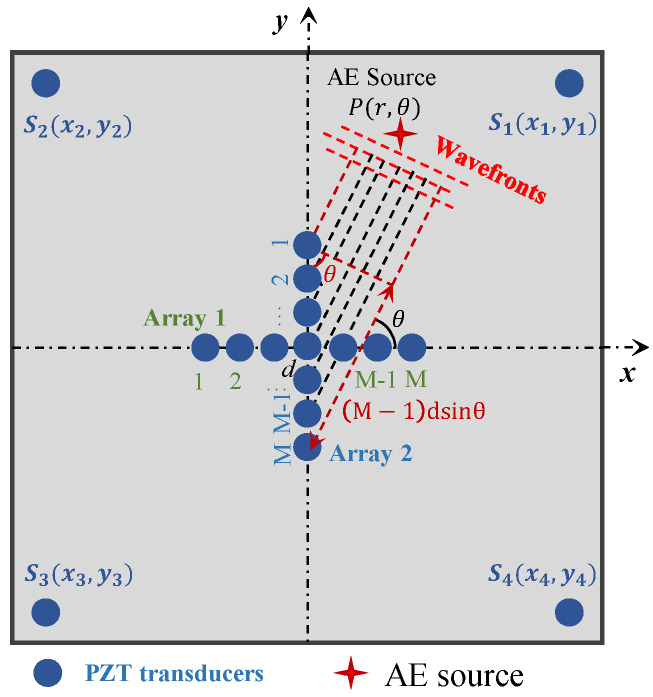
Illustration of wave velocity calculation.

In the proposed automatic wave velocity determination method, as depicted in Figure 2, sensor 1 and sensor M in Array 1 or Array 2 are used to calculate wave velocity; this is because the distance between these two sensors is the farthest and the difference of TOA between these two sensors is the largest. The basis for selecting Array 1 or Array 2 to perform wave velocity determination is as follows: if the localized angular coordinate of AE source calculated from Equation (6) is between −45° to 45° and 135° to 235°, array 1 is used for wave velocity calculation. For a far-field acoustic emission P(r,θ), as shown in Figure 2, the wave velocity can be calculated as Equation (7). If the localized angular coordinate of the AE source calculated from Equation (6) is between 45° to 135° and 235° to 315°, array 2 is used for wave velocity calculation, and the wave velocity can be calculated as Equation (8).

(7) $$c=(M-1)d\left|\frac{cos\theta }{\Delta t}\right|\;(\mathrm{for}\;\theta \in (-45{}^{\circ},\;45{}^{\circ})\;\mathrm{or}\;\theta \in (135{}^{\circ},\;235{}^{\circ}))$$

(8) $$c=(M-1)d\left|\frac{sin\theta }{\Delta t}\right|\;(\mathrm{for}\;\theta \in (45{}^{\circ},\;135{}^{\circ})\;\mathrm{or}\;\theta \in (235{}^{\circ},\;315{}^{\circ}))$$

where Δt is the ΔTOA between sensor 1 and sensor M or sensor N. It should be noted that the wave velocity c refers to the group velocity in the θ direction, and for isotropic materials, it is the full-field group velocity c. However, in the actual calculation condition, its value may be affected by the error of polar angle and the error of ΔTOA between the element 1 and M. Therefore, to ensure the rigor of the proposed method, the calculated wave velocity is named the quasi-velocity.

**Figure 3:**
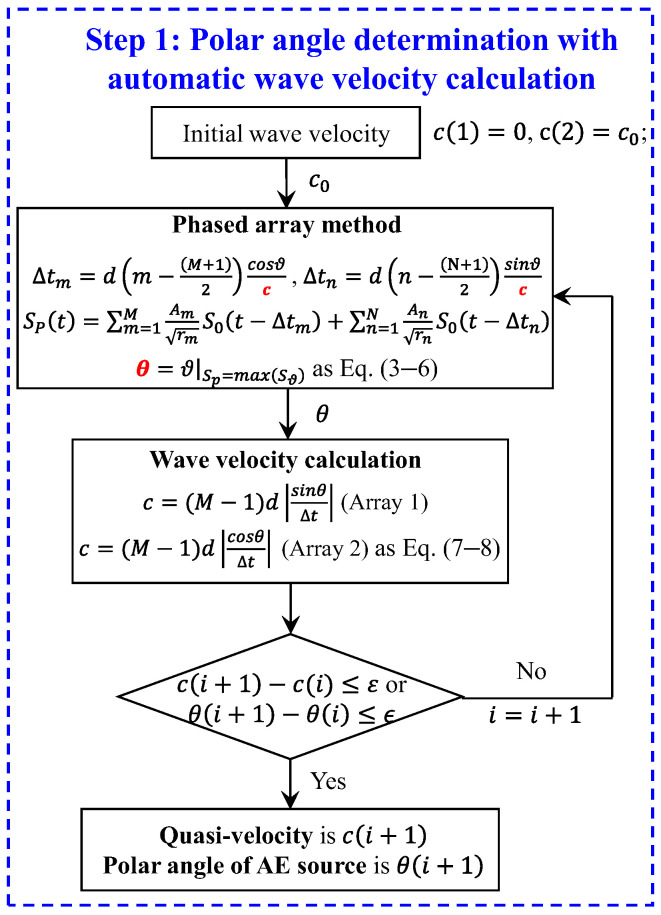
Flowchart of the iterative process to solve AE source polar angle.

The flowchart of iterative process to solve the AE source polar angle and quasi-velocity is shown in Figure 3. It can be found that the phased array method involves two unknowns, namely c and θ, as shown in Equations (3)–(8). Similarly, the process of wave velocity calculation also involves these two unknowns. Therefore, the polar angle and quasi-velocity of the acoustic emission source can be easily solved through iterations of the phased array method and the wave velocity calculation method. In the iterative process, a random value is given as the initial value $c_{0}$. With the initial wave velocity, the beamforming angle can be determined first. Using the calculated beamforming angle, the new wave velocity can be determined. The above steps are repeated until both of the two convergence criteria are satisfied, as shown in Equations (9) and (10). The quasi-velocity and angular coordinate are obtained. The convergence criteria of wave velocity and polar angle are as follows:

(9) $$c\left(i+1\right)-c(i)\leq \varepsilon$$

(10) $$\theta \left(i+1\right)-\theta (i)\leq \epsilon$$

where c is the calculated wave velocity, θ is the determined angular coordinate of AE source, ε and ϵ are the tolerances.

### 2.3. Location Determination

The radial distance is another important piece of information for the AE source localization method. For the passive GWPA method, determining the distance without knowing the value of TOF is difficult. To tackle problems of radial distance calculation in GWPA, four additional far-end transducers are added away from phased array transducers to provide enhanced information in step 2.

In a case where the polar angle of the AE source is known, any two sensors in the cross-shaped array that are not in the same line can theoretically be used to obtain the AE source position. However, in the actual condition, if the sensors are arranged very close to each other (e.g., the farthest distance is 3d in the proposed cross-shaped array), the unavoidable experimental error in calculating the TDOA will bring a large perturbation to the process of calculating the AE source. Therefore, four far-end sensors are innovatively arranged to eliminate the influence of the error of TDOA on the AE source localization results. The use of far-end sensors instead of sensors in the cross-shaped array is to increase the values of $x_{i}cos\theta +y_{i}sin\theta$ and $x_{i}^{2}+y_{i}^{2}-{\delta_{i}}^{2}$ in Equation (15). From the point of view of numerical analysis, the values of the numerator and denominator are increased at the same time, thus reducing the error of $\delta_{i}$ on the AE source localization results. It should also be noted that, when applying the proposed method in practical conditions, multiple sensor arrays may need to be used; then, the central sensor of one array may be used as the far-end sensor of another array, which means the far-end sensor will not additionally increase the number of sensors.

**Figure 4:**
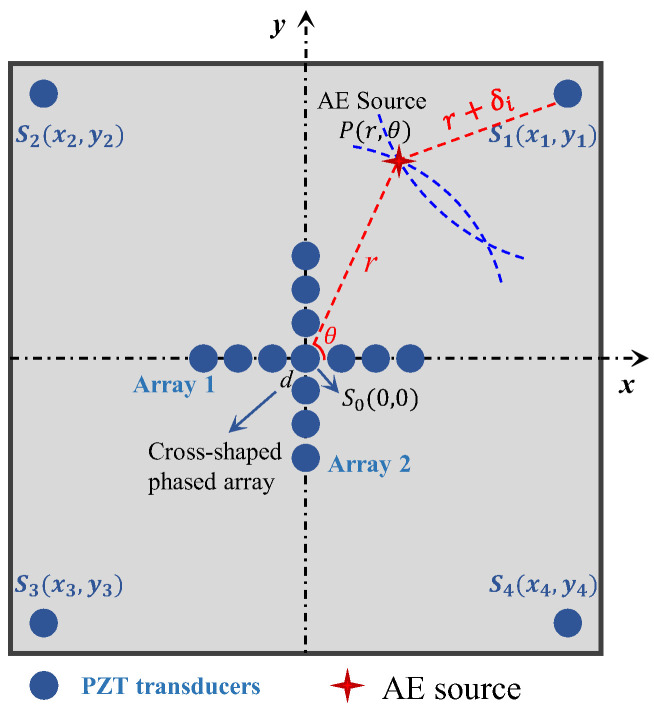
Method of location determination.

In the first step, the polar angle θ of the acoustic emission source and wave velocity c have been simultaneously determined by the iterative method. The quadrant where the acoustic emission source is located can be determined by the polar angle θ; furthermore, the far-end transducer in this quadrant will be utilized to provide an enhanced signal. In the second step, only two sensors are utilized to calculate the radial distance. Not to lose generality, we assume that one sensor is $S_{0}(0,0)$, and another sensor is $S_{i}(x_{i},y_{i})$ $\left(i=1,\;2,\;3,\;4\right)$, which is the enhanced transducer in the quadrant of the AE source. The acoustic emission source is P(x,y), with a distance r from $S_{0}$ and an angle θ from x axis, as shown in Figure 4. The equations of two circles are, respectively, as follows:

(11) $$x^{2}+y^{2}=r^{2}$$

(12) $${\left(x-x_{i}\right)}^{2}+{\left(y-y_{i}\right)}^{2}={\left(r+\delta_{i}\right)}^{2}$$

where $\delta_{i}$ is the difference in distance between the origin point $S_{0}$ and far-end point $S_{i}$ to the AE sources; the expression of $\delta_{i}$ is $\delta_{i}=\Delta t\cdot c$. ($\delta_{i}$ being positive means that point P is farther from the AE sources, and a negative value means that point P is closer to the AE sources).

Since the angle θ of the AE sources can be obtained first, the above equation can be expressed to polar coordinates as follows:

(13) x=rcosθ

(14) y=rsinθ

Therefore, the radial distance *r* is calculated as follows:

(15) $$r=\frac{x_{i}^{2}+y_{i}^{2}-{\delta_{i}}^{2}}{2\left(x_{i}cos\theta +y_{i}sin\theta +\delta_{i}\right)}$$

So far, a value of θ and a value of r can be derived, and the acoustic emission source is obtained uniquely.

**Figure 5:**
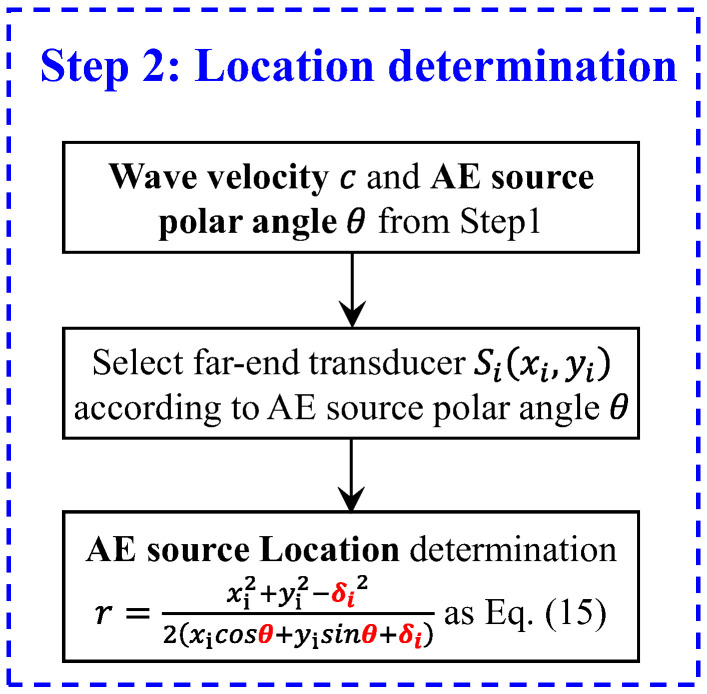
Flowchart of AE source location determination.

The flowchart of the implemented process to solve the AE source location is shown in Figure 5. In the first step, the wave velocity c and the AE source angular coordinate θ can be determined. In the second step, the AE angle coordinate will determine which enhancement transducer to use. Then, the radial distance of the AE source can be calculated by Equation (15).

Finally, the localization result of AE source is realized after using an image enhancement algorithm. The localization image can be obtained by displaying the energy of superimposed signals omnidirectionally. To distinguish the location of the AE source clearly, an imaging enhancement algorithm is proposed. The superimposed signals are normalized, and the exponential enhancement function is applied as shown below [28]:

(16) $$S^{\prime }\left(\theta ,t\right)={\left[\frac{S\left(\theta ,t\right)}{{S\left(\theta ,t\right)}_{max}-{S\left(\theta ,t\right)}_{min}}\right]}^{k}$$

where S is the amplitude of the superimposed signal before image enhancement, and $S^{\prime }$ is the enhanced signal amplitude, *k* is the power of exponential function, which controls the enhancement level compared to the original signals.

## 3. Numerical Study

### 3.1. Numerical Model

**Figure 6:**
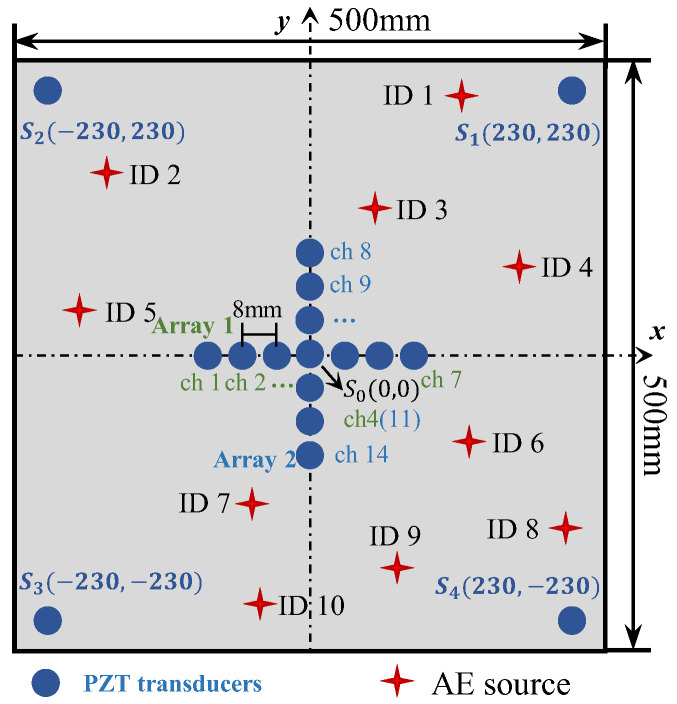
The location of sensors and AE sources in aluminum plate.

**Figure 7:**
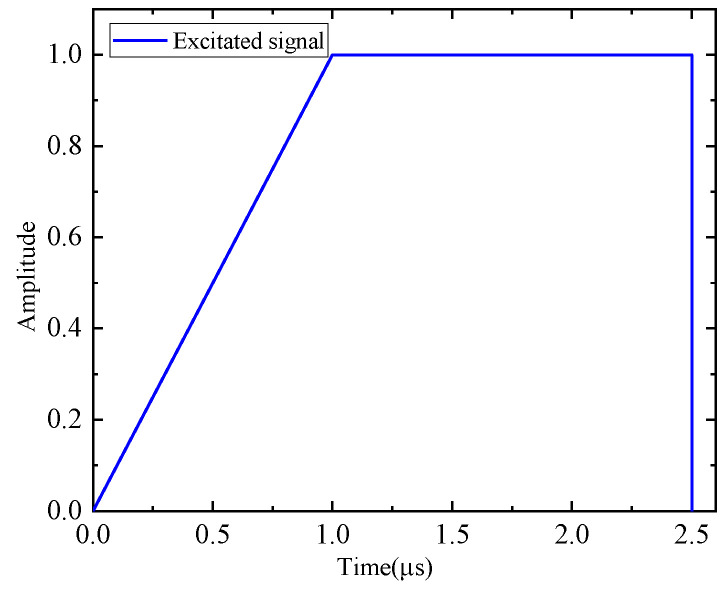
Simulated AE source function.

To validate the enhanced phased array method for acoustic emission source localization, a numerical study is first carried out. An aluminum plate with a dimension of 500 mm × 500 mm × 3 mm is considered, as illustrated in Figure 6. The properties of the material are Young’s modulus $E=7.1\times 10^{10}$ Pa, mass density ρ=2700 kg/m^3^ and Poisson’s ratio ν=0.33. In the center of the plate, a cross-shaped phased array with thirteen elements is placed on the top surface of the plate. Each linear array is made up of seven elements with 8 mm spacing between two adjacent elements. Four PZT elements are bonded near the boundary of the plate to provide enhanced signals, and the coordinates are shown in Figure 6. For the simulation of pencil lead break (PLB) AE events, a point load is applied on the top surface of the plate. A linear ramp function is used to simulate the AE signal, as shown in Figure 7 [43,44]. Simulated excitation positions are performed as shown in Table 1 and Figure 6.

The finite element tool ABAQUS is used for numerical modeling and analysis. To ensure high computational accuracy and efficiency, the element size and time step need to be reasonably selected [45]. The recommendation of maximum element size $l_{e}$ and time step $t_{e}$ suggested is shown in Equations (17) and (18). According to the frequency of the excitation signal, after mesh convergence analysis, the minimum element size is 0.5 mm and the time step is $1\times 10^{-8}\;s$.

(17) $$l_{e}=\frac{\lambda_{min}}{20}$$

(18) $$t_{e}=\frac{1}{20f_{max}}$$

where $l_{e}$ is the element length, $\lambda_{min}$ denotes the shortest interested wavelength and $f_{max}$ is the maximum excitation frequency.

### 3.2. Numerical Validation

Point ID 3 (135.5 mm, 67.5°) is introduced to illustrate the whole calculation process. The procedure of the enhanced phased array technique contains two steps. In the first step, the wave velocity and angular coordinate of the AE sources are solved simultaneously. To obtain the accurate wave velocity and the angular coordinate of the AE source, the iterative calculation is carried out repeatedly, as shown in Figure 3. When the wave velocity and angular coordinate of the AE source meet the convergence criterion in Equations (9) and (10), the iteration will terminate. After convergence verification, the convergence criterion is set to ε=200, ϵ=0.5. In the second step, the location of the AE source is determined.

**Figure 8:**
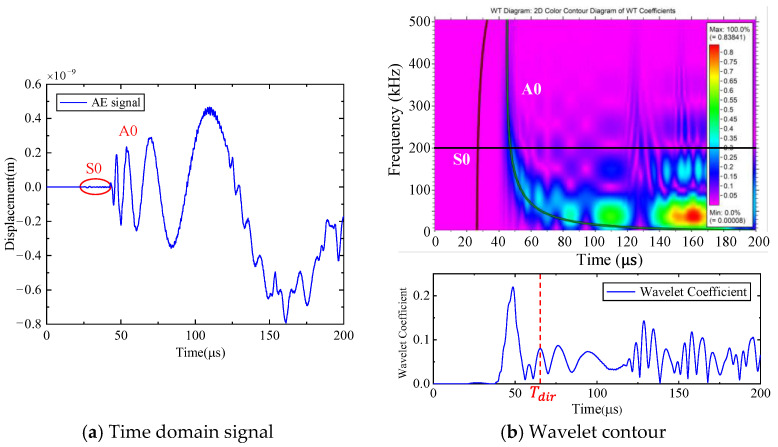
AE signal of ID 3 in the simulated calculation.

**Figure 9:**
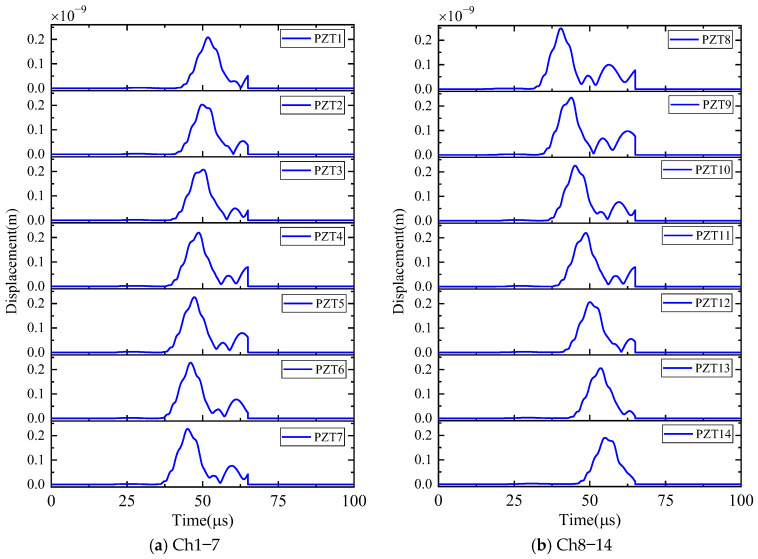
The AE signals of wavelet coefficients after CWT at frequency 200 kHz (simulated validation).

**Figure 10:**
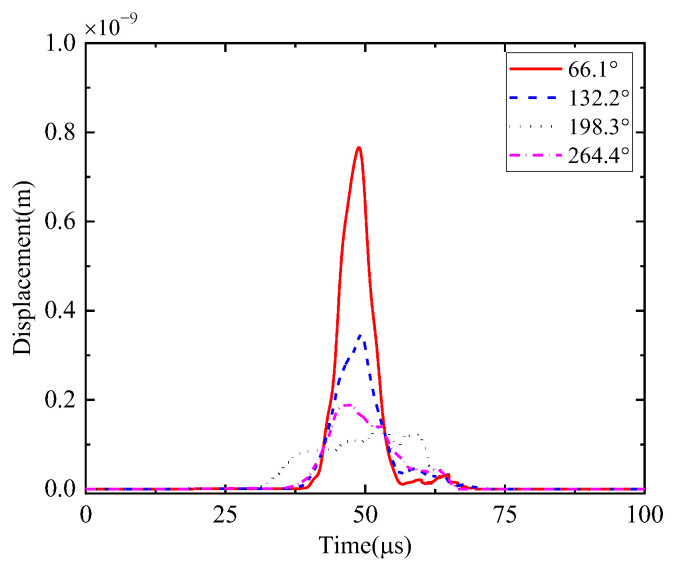
Superimposed signals in identified polar angle and other angles (simulated validation).

**Figure 11:**
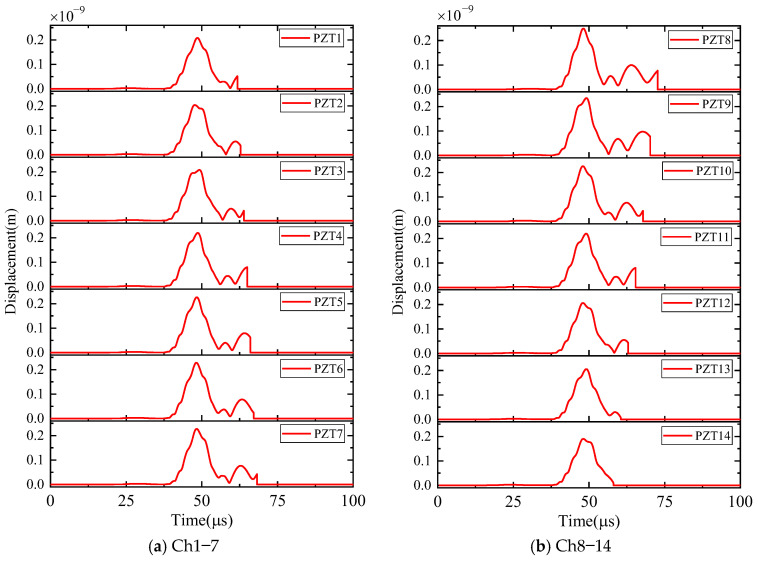
The shifted signals of wavelet coefficients at angle 66.1° (simulated validation).

First, the calculation process of the phased array algorithm is explained in the following section. The waveform of the signal acquired by ID 3 in channel 4 is shown in Figure 8a. Using Continuous Wavelet Transform (CWT) by the AGU-Vallen wavelet program [42], the received signals are transformed to obtain time–frequency domain signals. According to the cut-off frequency of Lamb wave and dispersion curves in Figure 8b, the AE wave only contains two modes, i.e., the A0 and S0 mode. The wave velocity of the S0 mode is larger than the A0 mode and the amplitude of the A0 mode is far greater than the S0 mode, so the A0 mode and S0 mode can be easily distinguished. The time window (0~$T_{dir}$), only including the direct A0 wave of the wavelet coefficient at 200 kHz, is used in the phased array algorithm properly, as shown in Figure 9. The theoretical wave velocity of the A0 mode in 200 kHz is 2993.0 m/s. The time delay of each transducer is calculated, respectively, as shown in Equations (3) and (4). All the signals are shifted by the calculated time delay from angle 0° to angle 360°; the interval angle is 0.1°. Then, the shifted signals are superimposed. Through shifting and superimposing the signals, the amplitude of the superimposed signal is greatly increased in the angular coordinate of the AE source. Finally, by comparing the amplitudes of the superimposed signals omnidirectionally, the AE source angular coordinate can be determined at angle 66.1°. Figure 10 shows the superimposed signal at angle 66.1° and other angles. It is found that the superimposed signal at 66.1° is much larger than that at other angles. Figure 11 exhibits the shifted signals at angle 66.1°. It can be clearly observed that the signals of all channels are shifted to the same phase, which proves the successful implementation of the phased array algorithm.

Then, the iterative processes are illustrated in this part. The initial wave velocity of the A0 mode is randomly given as 2000 m/s. In the first iterative step, the initial wave velocity of 2000 m/s is substituted into the phased array algorithm. The corresponding beamforming angle is calculated as 71.4°. In the second iterative step, the new wave velocity is solved as 2999.3 m/s using the above calculated polar angle. The corresponding polar angle is then calculated as 66.0°. In the third iterative step, the new wave velocity is solved as 3001.7 m/s. The corresponding polar angle is then calculated as 66.1°. This result meets the convergence criterion, and the iterative calculation terminates.

**Figure 12:**
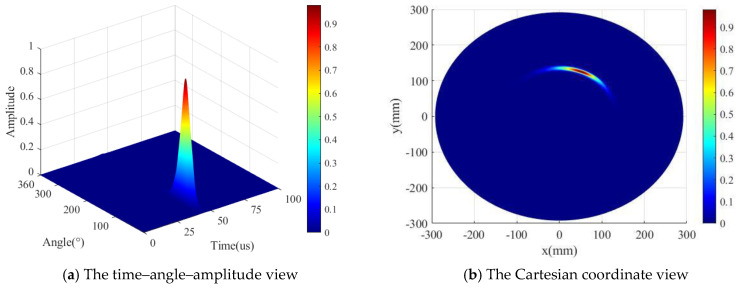
The detection result of AE source ID 3 (simulated validation).

In the second step, the location determination is explained. It can be concluded from the first step (the angular coordinate of AE source) that the AE source is located in the first quarter and the enhanced transducer $S_{1}\left(230,\;230\right)$ is utilized to precisely localize the AE source. The radial distance r can be ascertained according to Equation (15) using the sensors $S_{0}$ and $S_{1}$ along with the calculated polar angle. The TOA of the AE signal is determined at the maximum point of the direct A0 wavelet coefficient at 200 kHz. The localization result of ID 3 is shown in Figure 12; the localization result is strengthened using the enhancement algorithm as Equation (16), and the power of exponential function *k* is selected to be 5. Figure 12a shows the time–angle–amplitude result, which displays the TOF and the angle θ of the AE source. To observe the localization explicitly, Figure 12b shows the detection result in the Cartesian coordinate system. The detection result is (135.7 mm, 66.1°), and the real AE source location is (135.5 mm, 67.5°). The error of the polar angle is 1.4°, the location error is 3.3 mm, and the percentage error is 0.09%.

**Figure 13:**
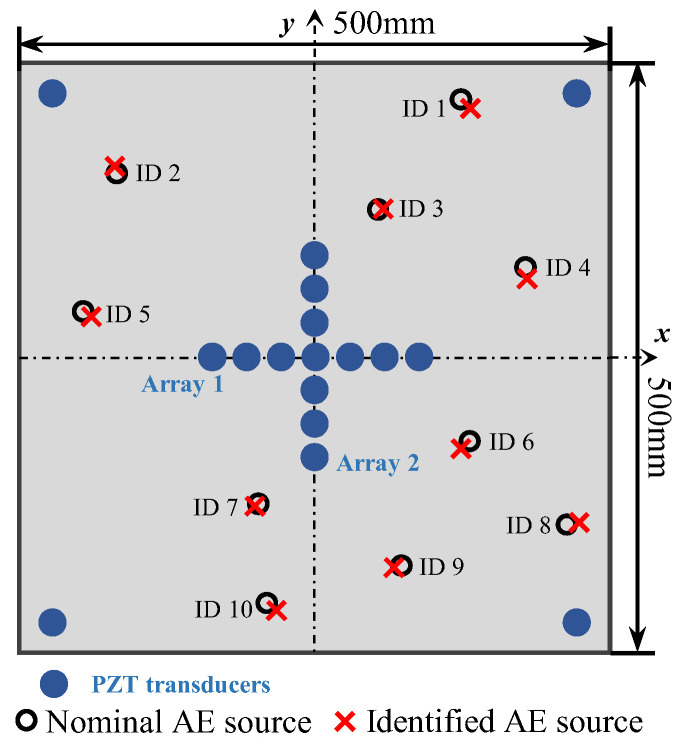
Localization results for different AE events (simulated validation).

Other concentrated forces are applied in the simulation model as shown in Table 1, the AE source localization results are shown in Table 2 and Figure 13. Because the simulation model provides high signal-to-noise ratio data, the error of localization result is small. The maximum error is 1.6% (No. 1) in all simulation points. Therefore, it can be concluded from Table 2 that accurate localization results can be achieved with full-range monitoring ability.

## 4. Experimental Study

### 4.1. Experimental Setup

**Figure 14:**
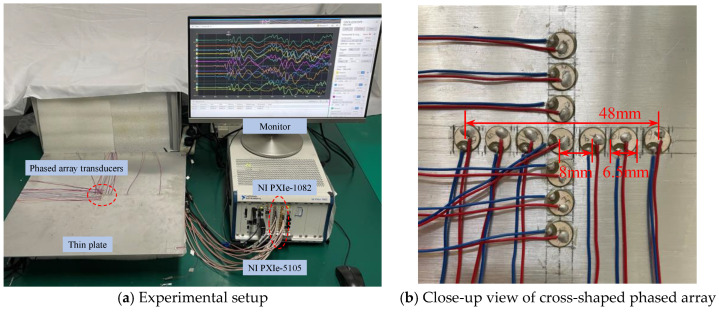
Experimental setup of the enhanced phased array method.

A National Instruments PXIe-1082 data acquisition system is adopted to record data from PZT elements bonded to the surface of the structure, as shown in Figure 14a. In this study, emphasis is placed on the data acquisition system and at least fourteen channels need to be output simultaneously. Two data acquisition cards (two PXIe-5105) are installed in the PXIe-1082 chassis to record data. The sampling rate is set as 60 MS/s. The trigger level of the experiment is 20 mV, the trigger channel is channel 4, i.e., the mid-point channel, and the total length of recorded time is 200 μs.

To evaluate the proposed localization methodology, a thin aluminum plate structure is introduced for experimental validation. The structure is made of aluminum alloy 6061, and the dimension is 500 mm × 500 mm × 3 mm, as shown in Figure 14a. In the center of the plate, a cross-shaped phased array of thirteen $d_{31}$-type PZT elements (P5-1) with a diameter of 6.5 mm and a thickness of 0.3 mm is attached to the top surface of the plate using 3M Scotch-Weld Epoxy Adhesives DP460, as shown in Figure 14b. Each linear array is made up of seven elements with 8 mm spacing between two adjacent sensors. Four PZT elements are bonded near the boundary of the plate and the coordinates can be seen in Figure 6. The AE events are simulated by the pencil lead break method using the fracture of mechanical pencil lead on the specimen surface, and the positions of the AE source are the same as in the simulated experiments. The CWT of the AE signals is calculated to obtain the wavelet coefficient. The TOA of the response signal is defined by the starting point of the A0 mode in the wavelet coefficient. To compare the accuracy of the proposed method with the classical triangulation technique, AE source localization experiments using the classical triangulation technique are also carried out. To validate the AE source localization ability for complicated structures, the proposed method is also extended to a stiffened thin-walled structure.

### 4.2. Experimental Validation

#### 4.2.1. Source Localization Test Using the Proposed Method

**Figure 15:**
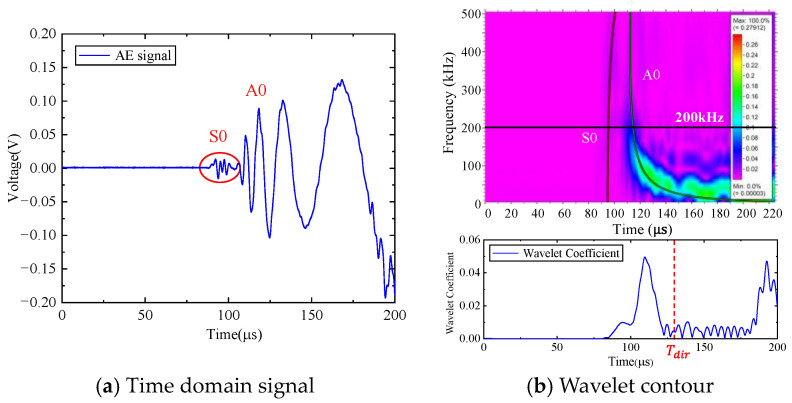
AE signal of ID3 in experiment.

The location of the AE events is the same as the simulation model, as shown in Figure 6. Table 1 shows the real coordinates of the AE sources. Experiment ID 3 (135.5 mm, 67.5°) is introduced here to illustrate the whole calculation process. The waveform of the signal acquired by ID 3 in channel 4 is shown in Figure 15a. Using Continuous Wavelet Transform by the AGU-Vallen wavelet program, the received signals are transformed to obtain time–frequency domain signals. The wavelet contour and wavelet coefficient received by ID 3 in channel 4 are shown in Figure 15b. The time window (0~$T_{dir}$) of the wavelet coefficient only including the direct A0 wave at 200 kHz is used in the phased array algorithm properly.

The procedure of the enhanced phased array technique contains two steps. In the first step, the wave velocity and angular coordinate of the AE sources are solved simultaneously. To obtain the accurate wave velocity and the polar angle, an iterative calculation is carried out repeatedly. When the wave velocity and angular coordinate of AE source meet the convergence criterion in Equations (9) and (10), which is ε=200, ϵ=0.5, the iteration will terminate. In the second step, the location of the AE sources is determined.

**Figure 16:**
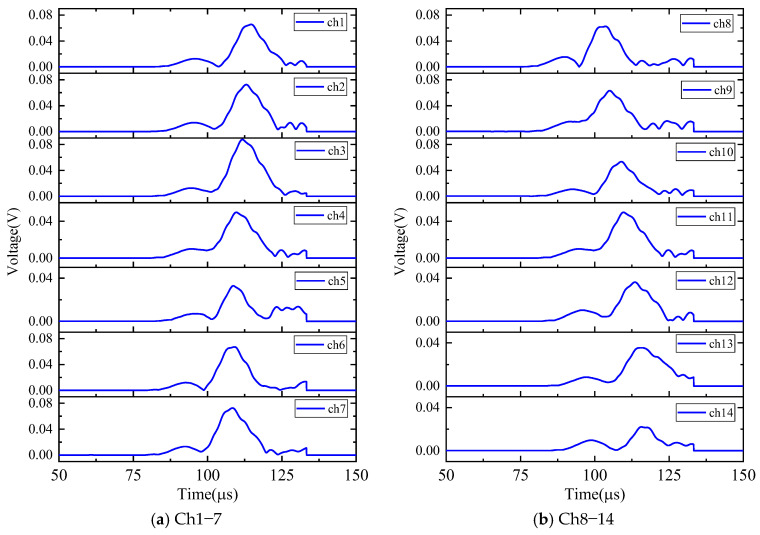
The AE signals of wavelet coefficients after CWT at frequency 200 kHz (experimental validation).

**Figure 17:**
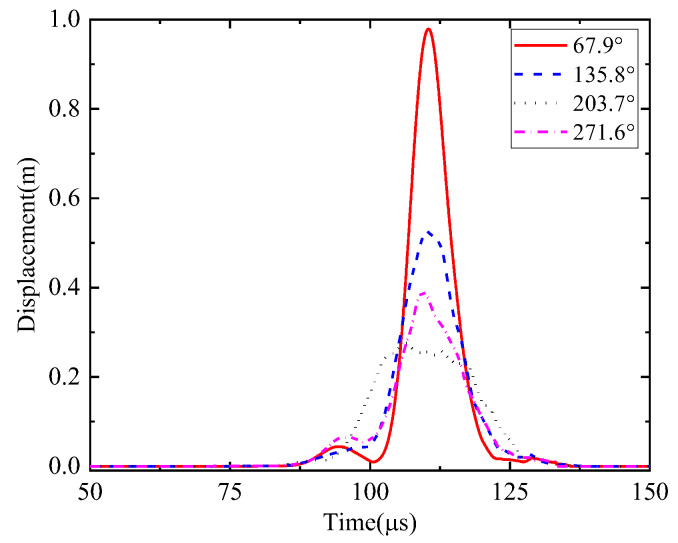
Superimposed signals in identified polar angle and other angles (experimental validation).

**Figure 18:**
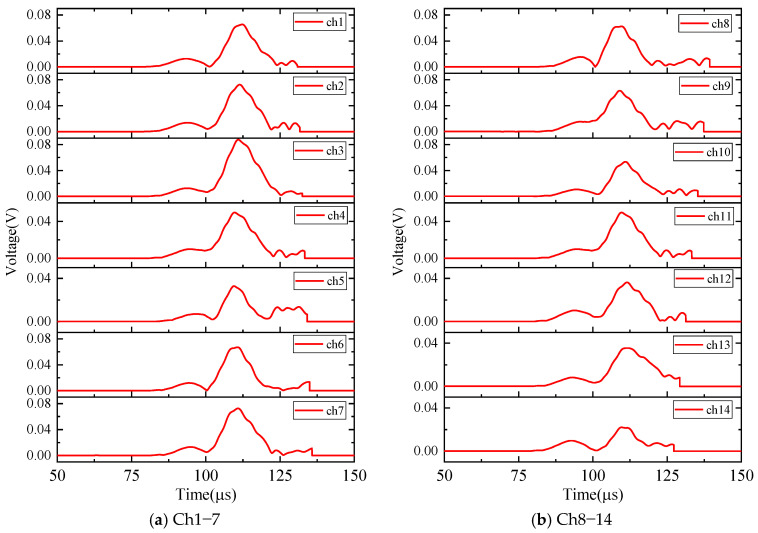
The shifted signals of wavelet coefficients at angle 67.9° (experimental validation).

The calculation process of the phased array algorithm is explained in this part. The time delay of each transducer is calculated, respectively, as Equations (3) and (4). The wavelet coefficient of the direct A0 mode is shown in Figure 16. All the signals are shifted by the calculated time delay from angle 0° to angle 360°, the interval angle is 0.1°. Then, the shifted signals are superimposed omnidirectionally. Through shifting and superimposing the signals, the amplitude of the superimposed signal in the angular coordinate of the AE source is greatly increased. Finally, by comparing the amplitude of the superimposed signals omnidirectionally, the angular coordinate of the AE source can be determined as angle 67.9°. Figure 17 shows the superimposed signal at angle 67.9° and other angles. It is found that the superimposed signal at 67.9° is much larger than that at other angles. Figure 18 exhibits the shifted signals. It can be clearly observed that the signals of all channels are shifted to the same phase, which proves the successful implementation of the phased array algorithm.

The iterative processes are illustrated in this part, as shown in Table 3. In the first iterative step, the initial wave is randomly given as 2000 m/s. Then, it is substituted into the phased array algorithm. The corresponding beamforming angle is calculated as 66.5°. In the second iterative step, the new wave velocity is solved as 3647.2 m/s using the above calculated polar angle. The corresponding polar angle is then calculated as 67.8°. In the third iterative step, the new wave velocity is solved as 3682.3 m/s. The corresponding polar angle is then calculated as 67.9°. The results meet the convergence criterion, then the iterative calculation terminates.

**Figure 19:**
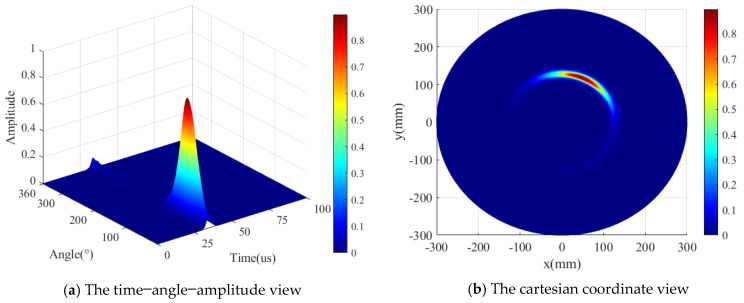
The detection result of the AE sources ID 3 (experimental validation).

In the second step, the location determination is explained. It can be concluded from the first step (the angular coordinate of the AE source) that the AE source is located in the first quarter, and the enhanced transducer $S_{1}\left(230,\;230\right)$ is utilized to precisely localize the AE source. The radial distance r can be ascertained according to Equation (15) using PZT $S_{0}$ and PZT $S_{1}$ along with the decided polar angle. The localization result of ID 3 is shown in Figure 19; the localization result is strengthened using the enhancement algorithm as Equation (16), the power of exponential function *k* is selected to be 5. Figure 19a shows the time–angle–amplitude result, which displays the TOF and angle θ of the AE source. To observe the localization explicitly, Figure 19b shows the detection result in the Cartesian coordinate system. The detection result is (127.7 mm, 67.9°), and the real AE source location is (135.5 mm, 67.5°). The angle error is 0.4°, the location error is 7.9 mm, and the percentage error is 1.1%. 

**Figure 20:**
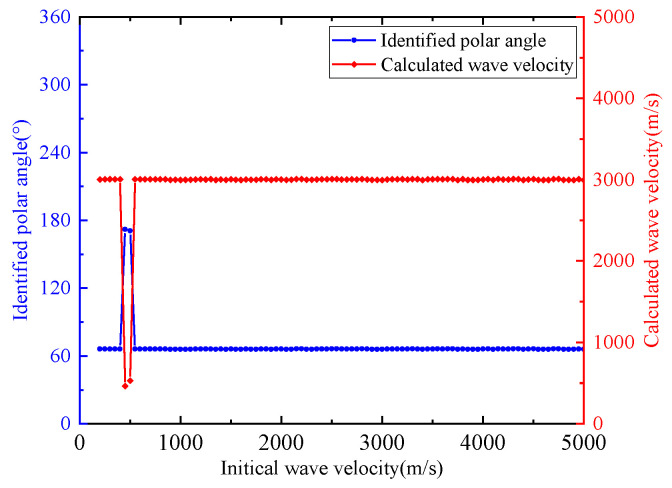
Initial wave velocity convergence analysis in experimental event ID 3.

It is also verified that the same wave velocity and identified polar angle can be obtained when the initial velocity changes a lot. Figure 20 shows the initial wave velocity convergence analysis of ID 3. When the initial velocity is given as 200–5000 m/s, accurate wave velocity and polar angle calculation can be realized. This indicates the stability and robustness of the proposed method; that is, in the case of any given initial velocity, the polar angle and wave velocity calculation can always converge to accurate results.

**Figure 21:**
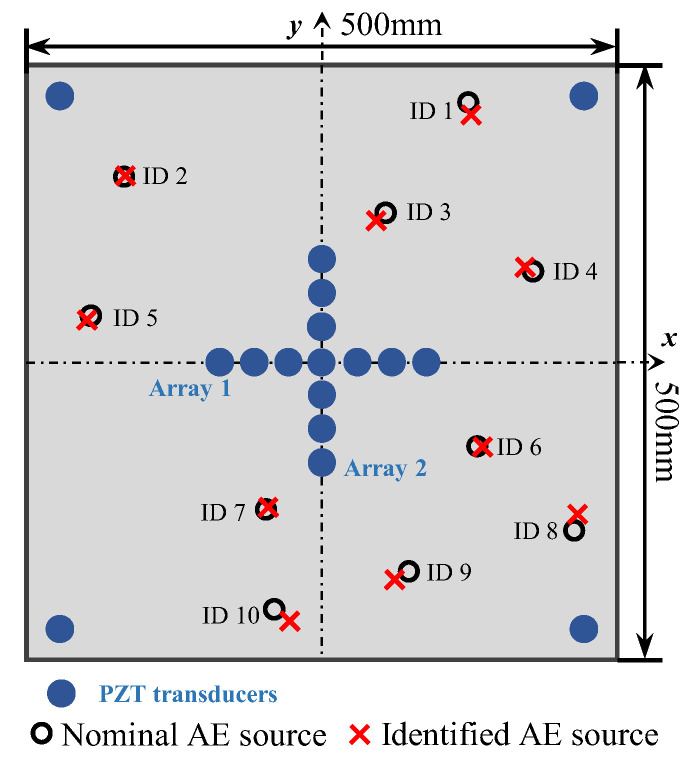
Localization results for different AE events (experimental validation).

Other experiments were performed and the results are shown in Table 4 and Figure 21; the maximum percentage error is 2.7%. The localization error of the AE source is slightly larger at the boundary, such as ID 1, ID 8, and ID 10, which is because of the aliasing of the direct wave and reflected wave of the A0 mode at the boundary. Therefore, the errors of polar angle and location result are relatively large, but still less than 3%. The results prove that the proposed method can accurately realize the AE source localization in all experiments, which also verifies the full-range monitoring of this method.

Analyzing the factors that affect the localization error can provide a basis for method improvement. In this paper, the localization discrepancy can be attributed to five aspects: Firstly, the wave pattern of AE signal is relatively complex and the frequency range is wide, which makes it difficult to locate the AE source. Secondly, since the phased array method is highly dependent on the array’s geometric configuration, this paper weighs the localization accuracy and the sensor number, and the 7-element cross-shaped phased array is selected. If the array configuration is further optimized, the localization accuracy may be improved. Thirdly, the proposed method is an under-information method; that is, the two unknowns are iteratively solved simultaneously, which may limit the localization accuracy to some extent. Fourthly, when the position of the acoustic emission source is close to the geometric boundary of the experimental piece, due to the influence of boundary reflection, the reflected wave and the direct wave may be aliased. The proposed method in this paper is based on the direct wave to determine the TDOA between the sensor channels, so the aliasing of the direct wave and the reflected wave will cause localization discrepancy. Fifthly, errors may be easily introduced during the experimental operation.

#### 4.2.2. Source Localization Test Using the Triangulation Method

**Figure 22:**
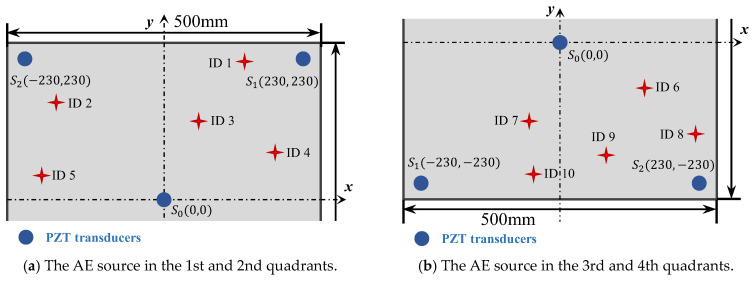
The location of sensors and the AE sources in the triangulation technique.

For comparison, the same experiments have been implemented using the classical triangulation technique. The locations of three sensors and AE sources are shown in Figure 22. The positions of the AE source are the same as in Table 1 to compare the localization results with the enhanced phased array method. In the triangulation technique, the AE source is determined by the differences in the TOA among three sensors. The determination of the TOA is consistent with the proposed method, which is the point of the maximum value in the direct A0 wavelet coefficient. The calculation process is consistent with the equation in Ref [15]. The wave velocity in the equations is set as 2993.0 m/s, which is the theoretical wave velocity of the A0 mode at 200 kHz.

**Figure 23:**
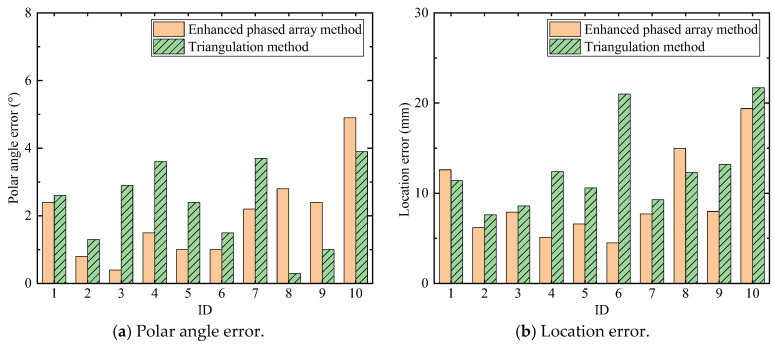
The comparison of localization results between the triangulation method and the enhanced phased array method.

The AE source localization results are shown in Figure 23. Figure 23a is the comparison result of the polar angle error between the triangulation method and the enhanced phased array method, and Figure 23b is the comparison result of the location error between these two methods. For all experiments, the localization results of the enhanced phased array method are nearly all better than the triangulation method. The average location error of the enhanced phased array method is 9.3 mm, and the average polar angle error is 1.94°. The average location error of the triangulation method is 12.81 mm, and the average polar angle error is 2.32°. Therefore, the proposed method can not only determine the wave velocity automatically, but its localization error is smaller than the classical triangulation method as well.

### 4.3. Experimental Application in Stiffened Plate

Stiffened thin-walled structures are widely utilized in aerospace engineering as critical load-bearing components [46]. These structures are prone to being damaged by external impact, corrosion, or fatigue cracks. Acoustic emission is a key phenomenon accompanying damage and can be used as an efficient approach to locating the damage in stiffened structures. However, due to the geometric complexity of the stiffened plate, the AE-resulted waves will be reflected and scattered continuously and therefore make the wave mode very complex, which may result in misleading information for most AE source localization methods. Under the circumstances, the enhanced phased array method is applied to the stiffened plate in this part to extend the application field of the method.

**Figure 24:**
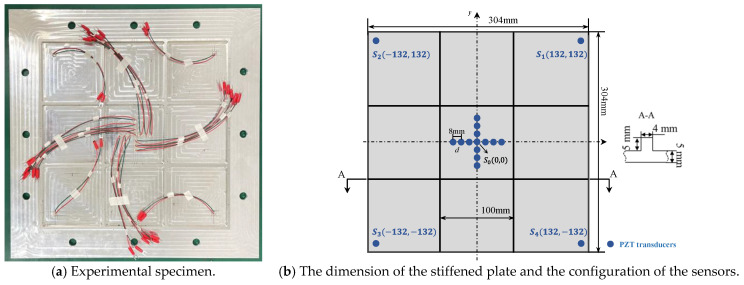
The overview of the stiffened plate.

The overview photograph of the stiffened plate is shown in Figure 24a, and the dimensions of the stiffened plate are shown in Figure 24b. The material properties of the host structure are Young’s modulus $E=7\times 10^{10}$ Pa, mass density ρ=2700 kg/m^3^ and Poisson’s ratio ν=0.3. The configuration of sensors is illustrated in Figure 24b. The same as the plate in Section 4.1, a cross-shaped phased array of thirteen $d_{31}$-type PZT elements (P5-1) is attached to the center of the stiffened plate. Each of the linear arrays of cross-shaped PZT array is made up of seven elements with 8 mm spacing between two adjacent arrays. Four PZT elements are bonded near the boundary of stiffened plate and the coordinates can be seen in Figure 24b. The AE events are actuated by a pencil lead break method, and the excitation positions are performed as shown in Table 5. The experimental parameters such as data acquisition system and sampling rate are the same in Section 4.1.

**Figure 25:**
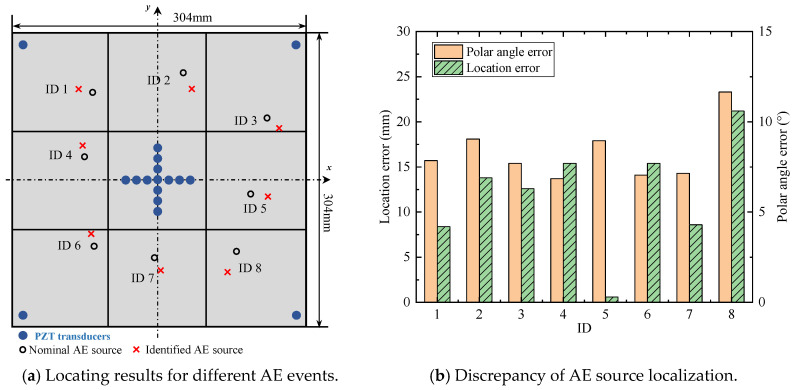
The AE source localization results of the stiffened plate.

The data processing method is completely consistent with Section 4.2. Since the thickness of the stiffened plate is slightly larger than the aluminum plate, the wavelet coefficient at 100 kHz is used in the phased array algorithm properly. The AE source localization results are shown in Figure 25. The maximum polar angle error is 10.6° and the maximum location error is 23.3 mm. Due to the complexity of the structure, the accuracy of the AE source location is relatively reduced. While the overall localization accuracy is basically within the acceptable range. Then, it can be proved that the proposed method can locate the AE sources in the stiffened plate correctly.

## 5. Discussions

In this section, the influences of several artificial parameters on the enhanced phased array method are studied in detail, in which the number of cross-shaped phased array elements and the time window length of AE signals are discussed. Moreover, the specimen and experiment setup are consistent with the example described in Section 4.1.

### 5.1. The Influence of Element Number

**Figure 26:**
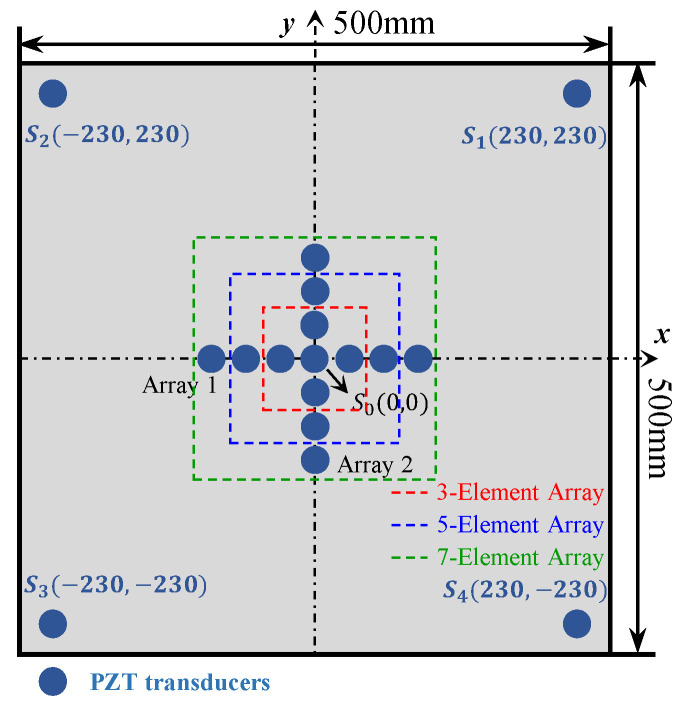
Configuration of different element numbers.

**Figure 27:**
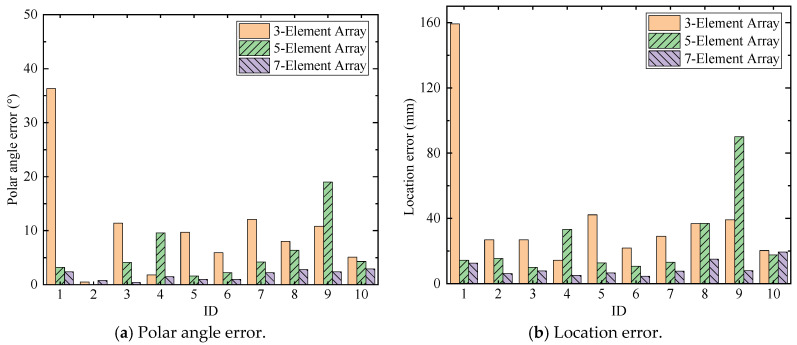
The comparison of localization results among 3-element array, 5-element array, and 7-element array.

The number of elements in the array is an important factor that affects the beamforming [47]. For the phased array method, with the number of array elements increasing, the beamforming effect and localization accuracy gradually improve. For the proposed automatic wave velocity determination method, according to Equations (7) and (8), as the distance between element 1 and element M increases, the influence of the error from ΔTOA on the wave velocity calculation result becomes smaller. However, in practice, more elements will result in wiring issues and the element number will be limited by the available transmitting channels. Thus, the influence of element number on the accuracy of AE source localization is studied in this part. The numbers of elements in a single array are 3-element array, 5-element array and 7-element array, respectively, as shown in Figure 26. The polar angle errors and location errors for all scenarios are shown in Figure 27.

From the experimental results in Figure 27, it can be found that the accuracy and robustness of AE source localization will improve with the increase in the array element number. For the 3-element or 5-element arrays, high accuracy can be achieved in some scenarios. But in some scenarios, localization distortion will occur, such as ID 1 in 3-element array and ID 9 in 5-element array. The polar angle and location results of 7-element (element number in the proposed method) array both maintain stable accuracy, as shown in Figure 27. This is because the sufficient number of array elements can improve the signal-to-noise ratio of the overall signals, thereby improving the stability and robustness of the proposed method. Therefore, considering the limitations of the equipment as well as the accuracy and stability of localization results, it is most suitable to choose a 7-element array for AE source localization using the enhanced phased array method, with a maximum polar angle error of 4.9° and a maximum location error of 19.4 mm (<3%).

### 5.2. The Influence of Time Window Length

**Figure 28:**
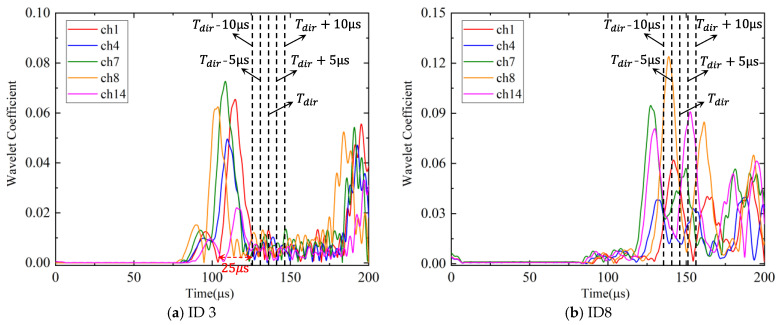
The schematic diagram of different time window lengths.

The time window 0~$T_{dir}$ that only includes the direct A0 wave of wavelet coefficient is used in the phased array algorithm properly, and $T_{dir}$ is defined as the arrival time that only includes the direct A0 wave in each experiment. The selected window length cannot be so short that it fails to contain the direct A0 wave of all channels, nor so long that it may contain reflected waves, resulting in inaccurate AE source localization results. Thus, the time window length $T_{dir}$ should be properly selected. In this section, different time window lengths are utilized in AE source localization. Since the time length of the direct A0 wave is about 25 μs, as shown in Figure 28, the lengths of the time window are increased or decreased by 20% and 40% of the direct wave wavelength. That is, the time window is selected as (0~$T_{dir}$-10 μs), (0~$T_{dir}$-5 μs), (0~$T_{dir}$), (0~$T_{dir}$+5 μs), (0~$T_{dir}$+10 μs).

**Figure 29:**
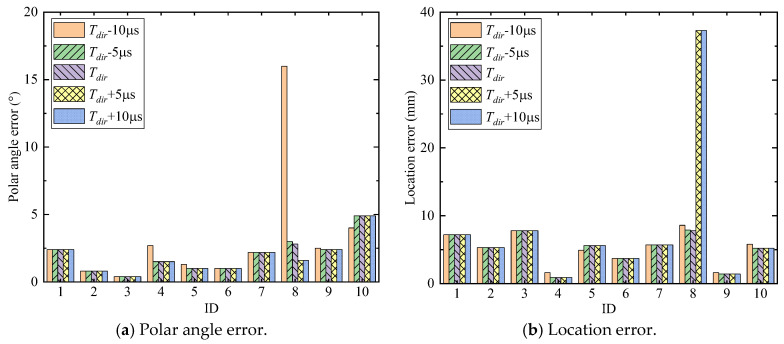
The results of localizing AE sources under different time window lengths.

Figure 28a is a schematic diagram of different time window lengths in ID 3. The wavelet coefficients of ch1, ch4, ch7, ch8, and ch14 of ID 3 are shown in Figure 28a, which are the most peripheral and central channels of the cross-shaped phased array. Since the difference in propagation time between the direct wave and the reflected wave in ID 3 is large, the five different window lengths selected do not affect the final result. The total localization results of experiments with different time window lengths are shown in Figure 29. For most experiments, the AE source localization results with different time window lengths are nearly consistent. For some experiments, when the selected window lengths are short, the time window fails to contain direct A0 waves of all channels, so the localization accuracy is slightly reduced, such as ID 4, ID 5, ID 8, ID 9, and ID 10. For experiment ID 8, after the window lengths increase by 20% and 40%, the polar angle error decreases slightly, but the location error increases significantly. The reason for this phenomenon is explained in detail below: the wavelet coefficients of ID 8 are shown in Figure 28b. It is found that after increasing the time window length, the TOA of channel 14 changes significantly (the maximum point in the selected time window), resulting in an inaccurate wave velocity and thus AE source location. The main reason for this phenomenon is that the location of the AE source in ID 8 is relatively close to the boundary, so it is more sensitive to the selection of the time window length. Based on the results, it can be concluded that the signal length indeed affects the results. From the general localization results in Figure 29, the proposed method is robust to a certain range of time window lengths.

## 6. Conclusions

This study presents an enhanced guided wave phased array method to better localize the AE sources in thin plates. Overall, the proposed method is a two-step method. In the first step, the polar angle is calculated by a cross-shaped phased array algorithm. An automatic wave velocity calculation method is developed, which can exactly determine the wave velocity and polar angle simultaneously. In the second step, the AE source location is determined via the help of a far-end sensor, decided polar angle and calculated wave velocity. Both numerical and physical experiment studies are carried out to validate the proposed AE localization method. The proposed method is also applied in a stiffened plate and the influences of artificial parameters are investigated. Several conclusions can be drawn from this study.

Firstly, compared with linear phased arrays, the proposed enhanced cross-shaped phased array can achieve 0~360° full range detection without ambiguities. It is proved that the proposed method can achieve high accuracy in any direction; that is, the proposed method solves the problem of blind area in the linear array effectively. Secondly, the enhanced cross-shaped phased array method can locate passive form damage with good accuracy. Passive form damage cannot know the TOF directly, so the radial distance localization using the phased array method becomes difficult. The enhanced cross-shaped phased array method uses an additional far-end sensor to solve this problem properly. Thirdly, the proposed method is applied to stiffened thin-walled structures with more complex geometries, and the results show that it can locate the AE events in the stiffened plate correctly. Finally, the effects of the phased array element number and the time window length of AE signals on the localization results are parametrically analyzed. Balancing the limitations of equipment as well as the accuracy of localization results, the 7-element array for the enhanced phased array method is very suitable. Based on the results of different time window lengths, it can be concluded that the proposed method is robust to a certain range of time window lengths.

## Figures and Tables

**Table 1:** Positions of simulated AE event.

Point ID	1	2	3	4	5	6	7	8	9	10
*x* (mm)	122.5	172.7	51.9	176.3	−200.0	129.7	−51.6	212.4	69.6	−43.5
*y* (mm)	218.2	154.9	125.2	76.3	38.6	−72.9	−125.9	−145.1	−178.0	−209.3

**Table 2:** The results of localizing the AE sources using the enhanced phased array method in the simulated calculation.

Point ID	Nominal Coordinates (r, θ) (mm, °)	Identified Coordinates (r, θ) (mm, °)	Quasi-Velocity (*c*) (m/s)	Polar Angle Error (*θ*) (°)	Location Error (*φ*) (mm)	Percentage Error
1	(250.2, 60.7)	(247.3, 58.2)	3044.4	2.5	11.2	1.6%
2	(232.0, 138.1)	(235.3, 137.3)	2953.9	0.8	4.7	0.7%
3	(135.5, 67.5)	(135.7, 66.1)	3001.7	1.4	3.3	0.5%
4	(192.1, 23.4)	(192.4, 20.4)	2363.2	3.0	10.1	1.4%
5	(203.7, 169.1)	(197.7, 170.0)	3806.0	−0.9	6.8	1.0%
6	(148.8, 330.4)	(144.4, 329.6)	3499.9	0.8	5.4	0.8%
7	(136.1, 247.5)	(136.8, 246.8)	3011.5	0.7	2.3	0.3%
8	(257.2, 325.7)	(262.6, 327.3)	2987.5	−1.6	9.2	1.3%
9	(191.1, 291.4)	(192.1, 290.0)	3053.8	1.4	4.6	0.7%
10	(213.8, 258.3)	(219.5, 260.6)	2980.2	−2.3	10.5	1.5%

**Table 3:** The iterative processes of ID 3.

	Wave Velocity (m/s)	AE Source Angular Coordinate (°)
Initial step	2000	
First iterative step	3647.2	66.5
Second iterative step	3682.3	67.8
Third iterative step	3669.0	67.9

**Table 4:** The results of localizing the AE sources using the enhanced phased array method in experimental validation.

Point ID	Nominal Coordinates (r, θ) (mm, °)	Identified Coordinates (r, θ) (mm, °)	Quasi-Velocity (*c*) (m/s)	Polar Angle Error (*θ*) (°)	Location Error (*φ*) (mm)	Percentage Error
1	(250.2, 60.7)	(243.0, 58.3)	3101.1	2.4	12.6	1.8%
2	(232.0, 138.1)	(226.7, 137.3)	2850.7	0.8	6.2	0.9%
3	(135.5, 67.5)	(127.7, 67.9)	3684.9	−0.4	7.9	1.1%
4	(192.1, 23.4)	(191.2, 24.9)	3207.3	−1.5	5.1	0.7%
5	(203.7, 169.1)	(198.1, 170.1)	3300.4	−1.0	6.6	0.9%
6	(148.8, 330.4)	(152.5, 331.4)	2640.5	−1.0	4.5	0.6%
7	(136.1, 247.5)	(130.4, 249.7)	3014.0	−2.2	7.7	1.1%
8	(257.2, 325.7)	(249.4, 328.5)	2801.7	−2.8	15.0	2.1%
9	(191.1, 291.4)	(192.5, 289.0)	3048.8	2.4	8.0	1.1%
10	(213.8, 258.3)	(219.0, 263.2)	2908.6	−4.9	19.4	2.7%

**Table 5:** Positions of AE event in stiffened plate.

Point ID	1	2	3	4	5	6	7	8
*x* (mm)	−69.4	25.0	112.0	−77.7	95.1	−67.1	114.5	165.3
*y* (mm)	89.1	110.4	63.4	22.5	−14.8	−69.7	−69.7	−55.7

## Data Availability

Data will be made available on request.
